# Impact of a Modified Version of Baby-Led Weaning on Infant Food and Nutrient Intakes: The BLISS Randomized Controlled Trial

**DOI:** 10.3390/nu10060740

**Published:** 2018-06-07

**Authors:** Liz Williams Erickson, Rachael W. Taylor, Jillian J. Haszard, Elizabeth A. Fleming, Lisa Daniels, Brittany J. Morison, Claudia Leong, Louise J. Fangupo, Benjamin J. Wheeler, Barry J. Taylor, Lisa Te Morenga, Rachael M. McLean, Anne-Louise M. Heath

**Affiliations:** 1 Department of Human Nutrition, University of Otago, PO Box 56, Dunedin 9054, New Zealand; liz.erickson@otago.ac.nz (L.W.E.); jill.haszard@otago.ac.nz (J.J.H.); liz.fleming@otago.ac.nz (E.A.F.); lisa.daniels@otago.ac.nz (L.D.); brittany.morison@gmail.com (B.J.M.); leocl333@student.otago.ac.nz (C.L.); louisejbee@gmail.com (L.J.F.); lisa.temorenga@otago.ac.nz (L.T.M.); 2 Department of Medicine, Dunedin School of Medicine, University of Otago, PO Box 56, Dunedin 9054, New Zealand; rachael.taylor@otago.ac.nz; 3 Department of Women’s and Children’s Health, Dunedin School of Medicine, University of Otago, PO Box 56, Dunedin 9054, New Zealand; ben.wheeler@otago.ac.nz; 4 Department of the Dean, Dunedin School of Medicine, University of Otago, PO Box 56, Dunedin 9054, New Zealand; barry.taylor@otago.ac.nz; 5 Department of Preventive and Social Medicine, Dunedin School of Medicine, University of Otago, PO Box 56, Dunedin 9054, New Zealand; rachael.mclean@otago.ac.nz

**Keywords:** Baby-Led Weaning, Baby-Led Introduction to SolidS, traditional spoon-feeding, complementary feeding, infants, nutrient intake, food intake

## Abstract

Despite growing international interest in Baby-Led Weaning (BLW), we know almost nothing about food and nutrient intake in infants following baby-led approaches to infant feeding. The aim of this paper was to determine the impact of modified BLW (i.e., Baby-Led Introduction to SolidS; BLISS) on food and nutrient intake at 7–24 months of age. Two hundred and six women recruited in late pregnancy were randomized to Control (*n* = 101) or BLISS (*n* = 105) groups. All participants received standard well-child care. BLISS participants also received lactation consultant support to six months, and educational sessions about BLISS (5.5, 7, and 9 months). Three-day weighed diet records were collected for the infants (7, 12, and 24 months). Compared to the Control group, BLISS infants consumed more sodium (percent difference, 95% CI: 35%, 19% to 54%) and fat (6%, 1% to 11%) at 7 months, and less saturated fat (−7%, −14% to −0.4%) at 12 months. No differences were apparent at 24 months of age but the majority of infants from both groups had excessive intakes of sodium (68% of children) and added sugars (75% of children). Overall, BLISS appears to result in a diet that is as nutritionally adequate as traditional spoon-feeding, and may address some concerns about the nutritional adequacy of unmodified BLW. However, BLISS and Control infants both had high intakes of sodium and added sugars by 24 months that are concerning.

## 1. Introduction

In conventional approaches to complementary feeding, parents typically spoon-feed their infant puréed foods, with gradual introduction of a wider variety of tastes and textures as their experience with solid foods develops. It is not until around 12 months of age that the infant is generally expected to be consuming the same foods as the rest of the family [1,2]. Baby-Led Weaning (BLW) is an alternative method for introducing solids where the infant feeds themselves all their foods from the start of the complementary feeding period. This self-feeding necessitates the use of handheld or finger foods as children of this age do not have the manual dexterity to use utensils to feed themselves puréed food [3]. Parents are also encouraged to include their infant in family meals [4]. As a result, BLW might be expected to result in differences in food, and therefore nutrient, intake at least in the early months of complementary feeding, and perhaps into childhood.

There is growing international interest in BLW with many parents using the approach and yet, surprisingly, we know almost nothing about the food and nutrient intake of infants who are following a baby-led approach to infant feeding. Retrospective questionnaire data indicate that different foods are offered as first foods. Infants following BLW are more likely to be offered fruit, vegetables, bread and rusks, whereas infant cereal (which would usually be fortified) is the main first food for conventionally fed infants [3,5,6,7]. In addition, concern has been expressed that energy and iron intakes may be inadequate and sodium intakes high [8]. However, only one study appears to have examined food intake using more rigorous prospective tools. Morison et al. [9] analyzed weighed diet records collected in a small sample of 6–8 month old infants following BLW (*n* = 25) or traditional spoon-feeding (*n* = 26). Despite similar energy intakes, the BLW infants were consuming less protein, carbohydrate and dietary fiber, and more fat (including saturated fat). Lower intakes of iron, zinc, calcium, vitamin B12 and vitamin C were also observed, although no difference was apparent in sodium intakes. Whether these differences persist at older ages has not yet been determined.

In response to concerns from health professionals that unmodified BLW might be associated with increased risk of growth faltering, iron deficiency and choking [10], the Baby-Led Introduction to SolidS (BLISS) randomized controlled trial investigated the impact of a modified form of BLW on these outcomes. We have shown that this modified version of BLW was associated with a number of benefits including: a reduction in food fussiness compared to traditional spoon-feeding [11]; higher iron [12] and zinc [13] intakes than have been reported for unmodified Baby-Led Weaning [9]; and no evidence of an increased risk of iron deficiency [12], choking [14] or growth faltering [11]. However, it is not known whether BLISS has beneficial or detrimental impacts on infant food and nutrient intake.

The aim of this paper was to determine the impact of a modified version of BLW, Baby-Led Introduction to SolidS (BLISS), on food and nutrient intake in infants from 7 to 24 months of age.

## 2. Materials and Methods

### 2.1. Subjects and Recruitment

Protocol [15], pilot study [16], and main outcomes [11] papers have been published for the BLISS study as a whole, so only information relevant to the current paper is provided here. The study was approved by the Lower South Regional Ethics Committee (LRS/11/09/037) and written informed consent was obtained from all adult participants before randomization. The study is registered with the Australian New Zealand Clinical Trials Registry (ACTRN12612001133820).

**Figure 1:**
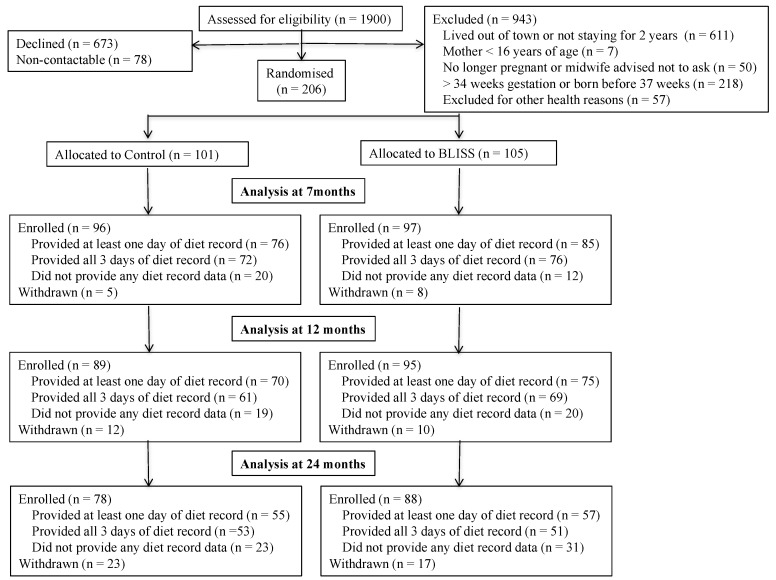
Flow of participants through the study.

Women were recruited in late pregnancy from sequential bookings (December 2012 to March 2014) at Queen Mary Maternity Hospital, the only maternity hospital serving Dunedin, New Zealand. The hospital has more than 97% coverage of births in the area. Women who were planning home births were invited to participate through their midwives. Exclusion criteria applied in late pregnancy were: not living locally, mother 16 years of age or younger, or booking with the maternity hospital after 34 weeks gestation. Further exclusion criteria applied after birth were: prematurity, or congenital abnormality likely to affect feeding or growth. The final sample size was 206 participants (Figure 1). Participants were randomly allocated to Control (*n* = 101) or BLISS (*n* = 105) groups using random length blocks after stratification for parity (first child vs. subsequent child) and maternal education (non-tertiary vs. tertiary).

### 2.2. Intervention

All families had access to routine government-funded midwifery (pregnancy to 4–6 weeks postnatal) and well-child (4–6 weeks to 5 years of age) care [17,18], and families in the BLISS group received 8 additional BLISS contacts (pregnancy to 9 months of age). An International Board Certified Lactation Consultant (IBCLC) provided education and support on delaying the introduction of complementary foods until 6 months of age, ideally by prolonging exclusive breastfeeding, during three face-to-face (antenatal, 1 week, and 3–4 months of age; 30–60 min each) and two telephone (3–4 weeks and 5 months of age; 10 min each) contacts. When the infant was 5.5, 7 and 9 months of age, each family met with a trained researcher for individualized advice and support about how to follow the BLISS approach to complementary feeding (30–60 min each) [15]. We used a variety of pretested [16] resources (recipe books, pamphlets, and information guides) to show parents how to adapt family meals to follow a baby-led philosophy, while ensuring sufficient iron and energy intakes, and limiting choking risk. In particular, we advised parents to offer at each meal: a “high iron” food (e.g., iron-fortified infant cereal or red meat), a “high energy” food (i.e., more than 6.3 kJ/g), and an “easy-to-eat” food (i.e., a food that was palatable and easy for infants to pick up). A resource was provided at each visit with safe age-appropriate examples of such foods. Resources also addressed how to: (1) wait until 6 months before introducing solids; (2) support any other people caring for the child to use a baby-led approach with the infant; (3) promote responsive feeding practices paying attention to hunger and satiety cues; (4) offer foods that are easy to pick up and eat; and (5) offer more frequent milk feeds during illness and recovery. An example of one of the resources can be found in our protocol paper [15].

### 2.3. Outcomes

Demographic information was obtained at baseline (late pregnancy) from hospital records (birth weight, infant sex, parity, and level of household deprivation [19]) and questionnaire (maternal education, employment status, ethnicity, and self-reported pre-pregnancy height and weight), after mothers had provided consent but before randomization to intervention group.

**Figure 2:**
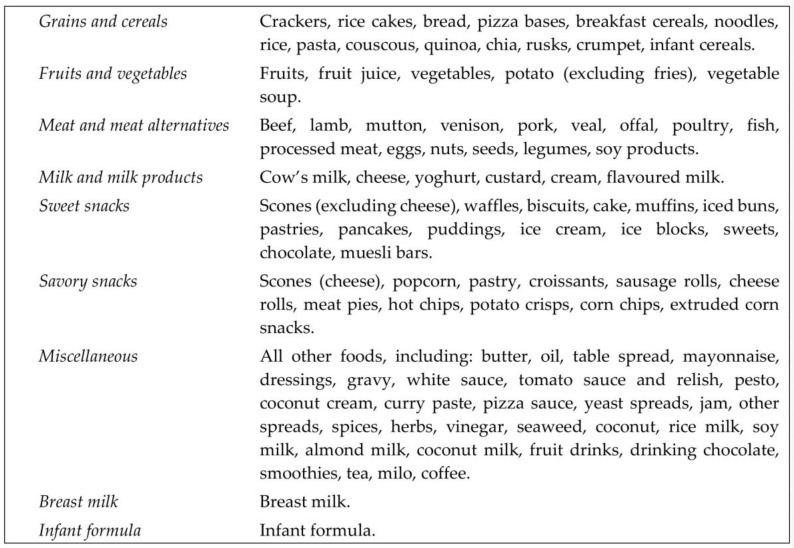
Descriptions of the nine food groups to which foods were assigned.

Three-day weighed diet records (3DDR) were collected on randomly assigned days (one weekend day, two week days) over a three-week period at 7, 12 and 24 months of age. Participants received extensive written and oral instructions for completing the records, and particular attention was paid to the measurement and recording of leftovers (including those on the floor, on clothes, etc.). For children consuming infant formula, parents were asked to record the weight of infant formula powder, added water, and total prepared weight of formula offered. Estimated total daily volumes were used for breast milk intake (750 g/day at 7 months [20], 448 g/day at 12 months [20], and 59 g/breastfeed at 24 months [21]). For infants who were mixed-fed, the reported volume of formula was subtracted from 750 g (7 months) or 448 g (12 months) as appropriate (no infant formula was consumed at 24 months). Data were analyzed using Kai-culator Version 1.13 s (University of Otago, Dunedin, New Zealand), which uses the New Zealand Food Composition Database (FOODfiles 2010), nutrient data for commonly consumed recipes from National Nutrition Surveys, and nutrient data for commercial infant foods [22] and added sugars data [23] collated by the research team. “Added sugars” were defined as mono- and di-saccharides added to foods by the consumer, cook or manufacturer as a sweetener and included sugars from syrups, honey and fruit concentrates, but not fruit juices and sugars from fruits in jams, jellies, preserves and spreads [23]. Each food or drink was assigned to one of nine food groups (Figure 2).

To assess adherence to a baby-led approach to complementary feeding, additional information was obtained for each food or beverage item recorded in the weighed food records: parents were asked to indicate *who* put each food or beverage item in the baby’s mouth (an adult, the child, or adult and child). For example, “adult and child” would be appropriate if a parent helped the child guide the spoon to their mouth, or both parent and child held a bottle of infant formula [15]. At the end of each day of diet recording, parents completed an “end of day questionnaire” where they indicated for each of the breakfast, lunch and evening meals: (1) whether the child ate with at least one other adult (yes/no); and whether the (2) meal ingredients and (3) meal preparation were similar to the family meal (four answer options for both: exactly the same, almost the same, similar, and mostly different).

### 2.4. Statistics

The primary aim of the BLISS study was to determine the impact of BLISS on body mass index (BMI) at 12 months of age, so the sample size calculations were based on detecting a difference in BMI (i.e., 85 infants were required in each group to detect a difference in BMI of 0.40 with 80% power). The results of that analysis are reported elsewhere [11]. The current analysis of food and nutrient intakes was of a secondary outcome (i.e., measurement of dietary quality [15]) so no separate sample size calculation was carried out. Statistical analyses were undertaken using Stata 14.2 (StataCorp, College Station, TX, USA) [24]. Nutrient intakes were adjusted for intra-individual variation to estimate “usual intake” using the Multiple Source Method (MSM) program [25]. All comparisons between intervention groups were adjusted for randomization strata (maternal education and parity) and infant sex. Baseline demographics were described (Table 1) and differences between mothers who provided diet records and those who did not were assessed with *t*-tests and chi-squared tests as appropriate.

Adherence to the BLISS approach was assessed by calculating the percent of daily food intake by weight (grams) consumed by the child that had been self-fed by the child, fed by an adult and the child, or fed by an adult (Table 2). For each category, the median (25th, 75th percentile) of the percentage of daily food intake by weight for each study group was reported and differences between the groups determined by median regression bootstrapped with 100 replications.

For the proportion of days that the child ate breakfast, lunch, and the evening meal: (1) with their family; (2) having the same (or almost the same) ingredients to the family meal; and (3) having the same (or almost the same) preparation to the family (Table 3), odds ratios and 95% confidence intervals were calculated using population-averaged generalized estimating equations so that each weighed diet record day was accounted for.

Geometric mean differences in nutrient intake between groups and 95% confidence intervals were calculated, and differences presented as percent difference of BLISS compared to Control (Table 4 and Table 5). Differences were assessed by regression analysis. Residuals were plotted and visually assessed for homogeneity of variance and normality, and influential outlier values investigated. Adequacy of nutrient intake was determined by comparing nutrient intake from the 3DDR with the Australian and New Zealand Nutrient Reference Values [26]; using the Estimated Average Requirement (EAR) Cutpoint method to determine the prevalence of inadequate intakes where an EAR was available, or else comparing mean group intake with the Adequate Intake (AI) [27]. The prevalence of excessive intakes of sodium was described by the prevalence of sodium intakes above the Upper Level of Intake (UL) at 12 and 24 months of age (no UL has been set for younger infants) [26,27]. The prevalence of higher than recommended intakes of added sugars was described by the prevalence of intakes equal to or greater than 5% energy, as is recommended by the World Health Organization [28].

**Figure 3:**
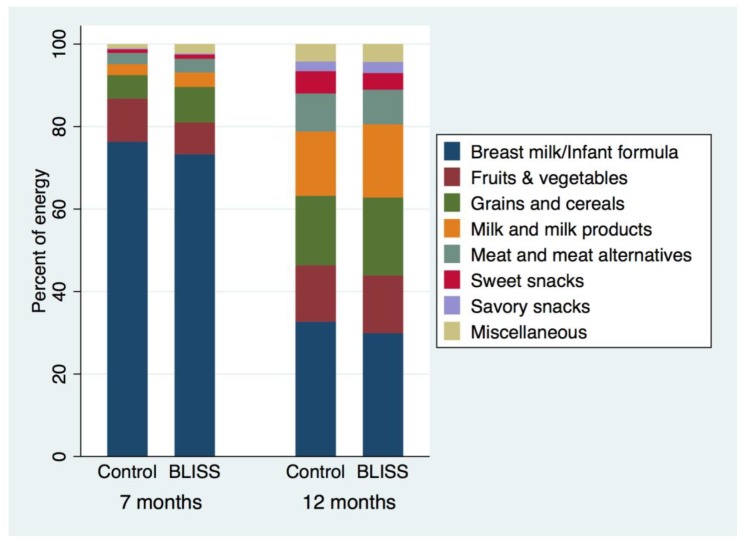
Mean proportion of energy from each food group at 7 and 12 months of age. See Figure 2 for definitions of the nine food groups that foods were assigned to.

Differences in food group energy intake between intervention groups were assessed by median regression at 7 and 12 months of age (Table 6 and Table 7). A bar graph was produced to illustrate the proportion of energy consumed from different food groups in the BLISS and Control groups at 7 and 12 months of age (Figure 3).

## 3. Results

In total, 206 mother–infant pairs were recruited into the study (24% of those eligible and contactable) (Figure 1). The mothers in the study were predominantly European (82%) and a high proportion (49%) were university educated (Table 1). Although they were less likely to be from deprived households (*p* = 0.07) than the mothers who were not recruited, they reported similar ethnicity and parity (*p* ≥ 0.12). Dietary data were provided by 161, 145, and 112 (83%, 79%, and 67% of enrolled) mother–infant pairs at 7 months, 12 months and 24 months of age, respectively. Women who provided at least one diet record for analysis (*n* = 165, 80%) were significantly older than those who did not (mean (SD): 32.1 (SD 5.2) compared with 28.2 (SD 6.0) years, *p* < 0.001), and were more likely to be University educated (52% compared with 34%, *p* = 0.039), but were similar in terms of maternal body mass index (BMI) (*p* = 0.426) and household deprivation (*p* = 0.945), and did not differ by study group (*p* = 0.508).

Infants in the BLISS group were exclusively breastfed for substantially longer (median 21.7 weeks; 95% CI: 13.0 to 23.8 weeks) than those in the Control group (median 17.3 weeks; 95% CI: 6.0 to 21.7 weeks; *p* = 0.002) [11]. In keeping with this, BLISS infants were introduced to solid foods later than Control infants (median 24.6 weeks compared to 22.6 weeks, *p* < 0.001) [12] and, therefore, fewer of them started solids before six months (26 weeks) of age (35% compared to 82%, *p* < 0.001).

At seven months of age, as a group, BLISS infants were feeding themselves on average 40% (25th, 75th percentile: 27%, 51%) of their food compared with Control infants who were feeding themselves on average only 9% (0%, 31%) of their food (*p* < 0.001) (Table 2). At 12 months of age, foods were slightly, but significantly, more likely to be fed by an adult in the Control group than in the BLISS group (7% vs. 0% of food; *p* = 0.027), but infants in both groups were feeding themselves virtually all of their food by 24 months of age. Infant involvement in family meals also differed between groups (Table 3). At seven months of age, BLISS infants were 2–4 times as likely to eat meals with their family (79% to 88% of meals compared to 61% to 75%), and to consume the same foods as the family (27% to 42% of foods compared to 9% to 22%), as Control infants. At 12 months of age, BLISS infants were still twice as likely to be eating the same foods as their family at lunch and evening meals (50% to 69% of foods compared to 22% to 55%).

There were no significant group differences in estimated intake of breast milk at 7 (difference BLISS relative to Controls, 95% CI: 0.0 g, −5.1 to 5.1; *p* = 1.00 [12]), 12 (−0.0 g, −0.1 to 0.1; *p* = 0.94 [12]) or 24 (−6 g, −32 to 20; *p* = 0.648) months of age. Corresponding differences (95% CI) in infant formula intake were 216 g (−97.2 to 530 g; *p* = 0.17) at 7 months and −85 g (−277 to 107 g; *p* = 0.38) at 12 months of age [12]. No infants were consuming infant formula at 24 months of age. BLISS and Control infants also consumed a similar weight of complementary food at each age (mean (SD) in BLISS vs. Control: 7 months 199 g (SD 177) vs. 233 g (SD 177), *p* = 0.293; 12 months 627 g (SD 339) vs. 588 g (SD 280), *p* = 0.393; 24 months 975 g (SD 348) vs. 920 g (SD 372), *p* = 0.367).

There were very few differences in nutrient intake between the BLISS and Control infants (Table 4 and Table 5). At seven months of age, BLISS infants ate significantly more total fat (percent difference, 95% CI: 6% more, 1% to 11% more) and sodium (35% more, 19% to 54% more) than Controls, but intakes of all other nutrients were similar. At 12 months, BLISS infants consumed significantly less saturated fat as a percentage of energy (7% less, 14% to 0.4% less) than Control infants. Nutrient intakes were similar for all nutrients at 24 months of age. There was no evidence of differences in nutrient adequacy between groups (data not shown): at seven months of age, intake appeared to be adequate for both groups for all nutrients measured (except total carbohydrate for which no conclusion can be made as the group mean intake was less than the AI [27], and dietary fiber for which no AI is defined). Although at 12 months of age 19% of infants had inadequate vitamin B12 intakes, and 15% had inadequate calcium intakes, by 24 months the intakes of just 4% and 6% of children, respectively, were inadequate.

As a group, the prevalence of excessive intakes of sodium and added sugars increased with age. Nine percent of infants had sodium intakes greater than the UL at 12 months (3% (*n* = 2) of Controls, 15% (*n* = 11) of BLISS; *p* = 0.018), but by 24 months, two-thirds (68%) of the children had excessive sodium intakes (66% (*n* = 37) of Controls, 70% (*n* = 40) of BLISS; *p* = 0.458). At seven months, 57% of infants consumed at least one sweet snack during the three days of diet recording, and 7% were already consuming 5% or more of their energy as added sugars (12% (*n* = 9) of Controls, 2% (*n* = 2) of BLISS; *p* = 0.013). By 12 months, 31% of children (39% (*n* = 27) of Controls, 24% (*n* = 18) of BLISS; *p* = 0.068) had intakes of added sugars that were higher than recommended, and this had increased to 75% by 24 months of age (80% (*n* = 45) of Controls, 70% (*n* = 40) of BLISS; *p* = 0.229).

Despite similar energy intakes, the sources of energy differed between BLISS and Control infants at seven months of age (Figure 3): “grains and cereals” contributed more energy to the diet in BLISS infants (difference, 95% CI: 127 kJ, 62 to 192 kJ), as did “meat and meat alternatives” (42 kJ, 8 to 77 kJ), “milk and milk products” (49 kJ, 17 to 80 kJ), and “miscellaneous foods” (32 kJ, 15 to 49 kJ, Table 6). By contrast, no significant differences in energy sources were observed at 12 months of age (Table 7).

## 4. Discussion

This randomized controlled trial demonstrates that a baby-led approach to introducing solids can meet nutrient requirements at seven months of age, although sodium intakes were higher in infants following Baby-Led Introduction to SolidS than in Controls. Total fat intake was slightly higher at 7 months, and percent energy from saturated fat slightly lower at 12 months, but by 24 months no differences were apparent for any of the nutrients measured. Similarly, intakes of “grains and cereals”, “meat and meat alternatives”, “milk and milk products”, and “miscellaneous foods” were significantly higher in the BLISS infants at 7 months, but by 12 months of age there was no evidence of differences in the food group intakes between infants following a baby-led approach and those following traditional spoon-feeding. Of concern, however, were the high intakes of sodium and added sugars in both groups by two years of age.

It is difficult to compare our findings with the literature given the scarcity of studies investigating food and nutrient intake in infants following a baby-led approach to complementary feeding. Only one small study (*n* = 51) has investigated the *nutrient* intake of infants following baby-led rather than traditional spoon-feeding approaches to complementary feeding, and only at 6–8 months of age [9]. Unlike the current study, Morison et al. [9] observed multiple differences in micronutrient intake between infants following BLW and their traditionally spoon-fed counterparts. Baby-Led Weaned infants consumed less iron (mean percent difference 59%), zinc (21%), calcium (19%), vitamin C (30%) and vitamin B12 (60%) than traditionally spoon-fed infants, although sodium intakes were comparable. In response to concern from health professionals that BLW might be associated with greater risk of growth faltering, iron deficiency, and choking [10], we made specific modifications to the diet recommended in our BLISS approach [15]. Presumably as a result of these modifications, BLISS infants appeared to have higher intakes of iron [12], and possibly zinc [13], vitamin C, vitamin B12 and calcium than have been reported for infants following unmodified BLW [9]. Importantly, intakes of iron [12], zinc [13], vitamin C, vitamin B12 and calcium were no lower than those of Controls (see discussion of the impact on sodium intake in the next paragraph). The nutritional adequacy of the BLISS approach to infant feeding was further supported by similar growth rates in BLISS and Control infants, and the lack of evidence of an increased risk of growth faltering in those following BLISS [11]. The earlier study of infants following unmodified BLW also reported significantly lower intakes of protein, carbohydrate, and dietary fiber, and higher intakes of total fat and saturated fat at 6–8 months of age [9]. Although the current study showed a similarly higher total fat intake as a gram amount, the contribution of fat to total energy intake was not significantly different (unmodified BLW infants consumed 15% more of their energy as fat than Controls (*p* < 0.01) [9], vs. BLISS infants consuming a non-statistically significant 1% (95% CI: −2% to 5%) more of their energy as fat than Controls). The only other macronutrient that appeared to be statistically significantly altered by BLISS was saturated fat (as a percentage of energy but not as a gram amount) which was slightly (7%) lower at 12 months of age. To date, no study has attempted to measure and report *food* intake in infants following BLW, although one small observational study reported that, somewhat surprisingly, infants following BLW were exposed to vegetables, fruit, carbohydrates, protein, “meals” and sweet foods less often than infants who were being spoon-fed [29]. Overall, BLISS appears to result in a diet that is as nutritionally adequate as traditional spoon-feeding, and may address some concerns about the nutritional adequacy of unmodified BLW.

However, sodium intake was significantly higher in infants following BLISS than in Controls at seven months of age. Dietary sodium intake is positively associated with blood pressure in adults [30] and children [31]. Furthermore, it has been suggested that if children develop a taste for salty food, it may carry on into adulthood leading to lifelong high sodium intakes [32]. Concern has been expressed that salt intake may be high in infants following unmodified BLW [8]—presumably because consuming family meals will lead to infants consuming additional sodium if the family food is salted—although the only other study that has attempted to measure sodium intake in infants following a baby-led approach to complementary feeding saw no difference in sodium intake between BLW infants and age- and sex-matched traditionally spoon-fed controls at 6–8 months of age [9]. The increased sodium intake of BLISS infants in the current study at seven months of age may be because parents were encouraged to use toast “fingers” as a vehicle for spreads such as iron-fortified infant cereal that they could not otherwise pick up themselves (to improve iron intakes), and also to offer cheese as an energy rich and palatable food (to minimize risk of growth faltering). Both foods are comparatively high in sodium. This suggestion is supported by the significantly higher intake of “grains and cereals” and “milk and milk products” in the BLISS group at seven months. However, the magnitude of the sodium difference at 7 months was not large (it was equivalent to around a ¼ slice of bread), and was not apparent at 12 and 24 months of age. This is in itself interesting as it suggests that, at least for these BLISS infants, even though they had higher intakes of sodium at 7 months, it did not result in them developing a greater preference for salty foods than Controls at 24 months. However, it is concerning that both groups showed a substantial increase in sodium intake with increasing age. In fact, although 9% of the study participants had sodium intakes at 12 months that exceeded the recommended UL (1000 mg/day), by 24 months of age this had increased to 68%. Few studies to date have reported the trajectory of sodium intakes prospectively in such a young cohort.

Parents have also been discouraged from adding any sugar to infants’ diets for many years, both because it is unnecessary and because it may increase liking of sweet foods [2]. More recently, the World Health Organization has recommended that free sugars should be less than 5% of energy intake in children and adults, in response to increasing international concern about the dose–response relationship between free sugars intake and dental caries (even in populations with water fluoridation) [28]. In the current study, BLISS and Control infants as a group had similar intakes of added sugars (similar to free sugars but excluding sugars from non-concentrated fruits in juices, jams, jellies, preserves and spreads) at 7, 12 and 24 months of age, and sweet snacks were being consumed in very small amounts by both groups at 7 and 12 months. However, 57% of infants had consumed at least one sweet snack during the three days of diet recording at seven months of age, so foods containing added sugars were being offered to many infants, despite Ministry of Health recommendations [2]. In addition, 7% were already consuming 5% or more of their total energy intake energy as added sugars. By 12 months, the number with higher than recommended intakes had increased to one in three children, and by 24 months, to three in four. Although BLISS infants were less likely than Controls to have excessive intakes of added sugars at 7 months, with a similar tendency at 12 months, the absolute numbers were 9 Controls and 2 BLISS infants at 7 months, and the difference was no longer apparent by 24 months. To our knowledge, these are the first longitudinal data published on added sugars intakes in infants and toddlers. They show a concerning trend with children already consuming an average of two teaspoons of added sugars a day (7 g in Controls) by one year and four teaspoons (17 g) by two years of age.

In addition to differences in nutrient and food intake between the two groups, particularly at 7 months of age, there were substantial differences in the way in which foods were fed. Infants in the BLISS group were substantially more likely to feed themselves (they self-fed 40% of their foods, compared to 9% for Controls), and were considerably more likely both to eat meals with their family, and to eat the same foods as their family. The only other study to collect data on individual foods and meals at the time of consumption reported even higher levels of self-feeding in 6–8-month old infants following unmodified BLW (77% of foods self-fed) [9], although the inclusion of eight month old infants in that study at least partially explains the higher rate of self-feeding. In keeping with our findings, high rates of family meal consumption have been widely reported in infants following BLW [5,7,9]. In fact, parents commonly report that the family being able to eat together is one of the advantages of BLW [6,10,33,34]. It is not clear what the health and social effects of self-feeding and participating in the family meal in infancy might be, but family meal involvement in children and adolescents is associated with consumption of healthier diets, and a reduced risk of overweight [35].

This study has several strengths including the randomized controlled design, rigorous assessment of dietary intake at multiple time points, and high levels of adherence. We took care to teach parents the importance of accurately measuring foods that were offered but not eaten (i.e., “leftovers”), given this is a particular challenge in this age group. We believe that parents did indeed record actual intake and uneaten foods well, a belief supported by median energy intakes reflecting current Estimated Energy Requirements [26]. We carefully assessed adherence to ensure that any lack of effect (if observed) was not a result of poor adherence given the randomized trial design. Who fed the infant was assessed for each individual food and drink item consumed over the three days of diet recording, and parents detailed whether each meal was the same as, or almost the same as, that consumed by the rest of the family, and whether meals were eaten together; all important components of a baby-led approach to feeding [4]. Indeed, these data revealed that substantially more foods were self-fed by infants in the BLISS group, and that BLISS infants were considerably more likely to consume meals with their families, and eat the same foods, than Controls.

The study also has some limitations. The sample was relatively small and as such should be treated as exploratory. We have reported confidence intervals to demonstrate the range of plausible values for the differences. Power calculations for energy and nutrient intake were not performed as the primary outcome of the study was to determine differences in child growth [11]. Not all parents completed diet records (20% provided none) and these participants were significantly younger and less well educated than parents who did provide dietary data, although the degree of household deprivation and maternal BMI were comparable. Overall, our BLISS study recruited a relatively socio-economically advantaged group, meaning that our findings may not apply to other groups, although 21% came from households with a high level of deprivation. Finally, we had to estimate breast milk volume which will have introduced some error, and future studies should endeavor to use more accurate measures of intake such as the deuterium oxide dilution “dose-to-mother” technique [36] where feasible. There is, however, no reason to believe that the estimates of breast milk intake differentially influenced the BLISS intake data because, although the BLISS group breastfed exclusively for longer, they were no more likely to be breastfeeding than Controls at 7 months of age, and there was no evidence of a difference between groups in the amount of complementary foods eaten.

In conclusion, BLISS appears to result in a diet that is as nutritionally adequate as traditional spoon-feeding, and may address some concerns about the nutritional adequacy of unmodified BLW. However, BLISS and Control infants both had high intakes of sodium and added sugars by 24 months that are concerning.

## Figures and Tables

**Table 1:** Characteristics of the study participants.

	Category	Control (*n* = 101) *n* (%)	BLISS (*n* = 105) *n* (%)
***Maternal variables***			
Age (years) ^1^	*Mean* (*SD*)	31.3 (6.2)	31.3 (5.0)
Parity	Primiparous	42 (41.6)	43 (41.0)
	Multiparous	59 (59.4)	62 (59.0)
Ethnicity	NZEO ^2^	85 (84.2)	83 (79.0)
	Māori or Pasifika	10 (9.9)	15 (14.1)
	Asian	6 (5.9)	7 (6.7)
Education	School only	29 (28.7)	34 (32.4)
	Post-secondary	19 (18.8)	24 (22.9)
	University	53 (52.5)	47 (44.8)
Pre-pregnancy BMI ^3^	*Mean* (*SD*)	25.6 (5.6)	25.9 (6.3)
***Household variables***			
Household deprivation	1–3 (Low)	29 (28.7)	31 (29.5)
	4–7	49 (48.5)	53 (50.5)
	8–10 (High)	23 (22.8)	21 (20.0)
***Infant variables***			
Age at 7mo measure (months) ^4^	*Mean* (*SD*)	7.2 (0.2)	7.2 (0.1)
Sex ^1^	Male	53 (53.0)	43 (41.0)
	Female	47 (47.0)	62 (59.0)
Ethnicity ^5^	NZEO ^2^	70 (69.3)	75 (71.4)
	Māori or Pasifika	23 (22.8)	22 (21.0)
	Asian	8 (7.9)	8 (7.6)
Birth weight (g) ^6^	*Mean* (*SD*)	3534 (490)	3517 (439)
Gestation at birth ^1^ (weeks)	*Mean* (*SD*)	39.7 (1.3)	40.0 (1.0)

Data expressed as *n* (%) except where indicated. Data missing for 1 ^1^, 7 ^3^, 25 ^4^, and 8 ^6^ participants; ^2^ NZEO refers to New Zealand European and Others; ^5^ Because the infants were too young to identify their own ethnicity, infant ethnicity was defined as either parent reporting their own ethnicity as other than New Zealand European at baseline. The following prioritization order was used when multiple ethnicities were reported: Māori or Pasifika, then Asian, then NZEO.

**Table 2:** Adherence to a baby-led approach to complementary feeding (using 3DDR data). ^1.^

	7 Months			12 Months			24 Months		
	Control (*n* = 77)	BLISS (*n* = 85)	*p* ^2^	Control (*n* = 69)	BLISS (*n* = 75)	*p* ^2^	Control (*n* = 56)	BLISS (*n* = 57)	*p* ^2^
Fed by an adult	27 (6, 44)	2 (0, 19)	**<0.001**	7 (0, 15)	0 (0, 9)	**0.027**	0 (0, 0)	0 (0, 0)	-
Fed by adult & child	52 (25, 64)	44 (27, 57)	0.262	21 (5, 36)	20 (9, 33)	0.771	0 (0, 11)	0 (0, 5)	>0.999
Self-fed by child	9 (0, 31)	40 (27, 51)	**<0.001**	59 (44, 71)	67 (49, 79)	0.109	89 (72, 99)	96 (88, 100)	0.060

Bold text indicates *p* < 0.05. ^1^ Data presented as median (25th, 75th percentile) percent of daily food intake by weight during the 3DDR; NB: Totals do not add to 100% because data are presented as medians; ^2^ Median regression bootstrapped with 100 replications, adjusting for maternal education and parity, and infant sex.

**Table 3:** Relationship between the foods eaten by the infant and the meals eaten by their family (i.e., family meals).

	7 Months			12 Months			24 Months		
	Control (*n* = 70)	BLISS (*n* = 82)	OR ^2^ (95% CI)	Control (*n* = 65)	BLISS (*n* = 72)	OR ^2^ (95% CI)	Control (*n* = 54)	BLISS (*n* = 56)	OR ^2^ (95% CI)
*Number of infants eating their meal with the family* ^1^									
Breakfast	41/55 (75%)	56/64 (88%)	**2.2 (1.0, 4.8)** ^3^	54/61 (89%)	64/67 (96%)	1.6 (0.6, 3.9)	48/53 (91%)	49/54 (91%)	1.8 (0.6, 5.4) ^11^
Lunch	33/54 (61%)	54/68 (79%)	**3.9 (2.0, 7.5)** ^4^	46/57 (81%)	54/63 (86%)	1.0 (0.5, 2.2) ^9^	38/47 (81%)	46/49 (94%)	**2.6 (1.1, 6.2)** ^5^
Evening meal	41/63 (65%)	67/78 (86%)	**4.5 (2.3, 8.6)** ^5^	48/58 (83%)	59/64 (92%)	1.8 (0.8, 4.1) ^10^	48/51 (94%)	48/50 (96%)	0.6 (0.2, 1.9) ^11^
*Number of infants with ingredients the same as the family meal* ^1^									
Breakfast	8/52 (15%)	19/65 (29%)	**3.4 (1.8, 6.6)** ^6^	29/59 (49%)	44/66 (67%)	1.4 (0.8, 2.5) ^11^	34/52 (65%)	40/55 (73%)	1.4 (0.8, 2.8) ^11^
Lunch	5/54 (9%)	17/63 (27%)	**2.5 (1.3, 4.8)** ^7^	12/54 (22%)	32/64 (50%)	**2.0 (1.1, 3.7)** ^10^	22/45 (49%)	27/49 (55%)	1.7 (0.9, 3.1) ^13^
Evening meal	14/63 (22%)	32/76 (42%)	**3.9 (2.1, 7.1)**	30/55 (55%)	45/65 (69%)	**2.1 (1.2, 3.8)** ^10^	42/51 (82%)	45/53 (85%)	1.3 (0.6, 2.6)
*Number of infants with meal preparation the same as the family meal* ^1^									
Breakfast	9/50 (18%)	25/63 (40%)	**3.5 (1.8, 6.6)** ^8^	33/57 (58%)	49/64 (77%)	**1.9 (1.1, 3.5)** ^9^	38/51 (75%)	43/55 (78%)	1.2 (0.6, 2.4) ^11^
Lunch	9/52 (17%)	17/59 (29%)	**2.2 (1.2, 4.0)** ^4^	19/52 (37%)	39/63 (62%)	**2.0 (1.1, 3.7)** ^12^	29/44 (66%)	34/48 (71%)	2.0 (1.0, 3.9) ^12^
Evening meal	11/61 (18%)	33/76 (43%)	**3.8 (2.1, 7.0)**	32/54 (59%)	47/65 (72%)	**2.1 (1.2, 3.9)** ^10^	45/49 (92%)	45/52 (87%)	1.1 (0.5, 2.5)

Bold text indicates *p* < 0.05. ^1^ Summary numbers are presented for the first day of the diet record for those infants who had that meal. The “same as” was defined as the participant answering 1 = exactly the same, or 2 = almost the same, on a four-point scale (other values were 3 = similar, 4 = mostly different); ^2^ Odds ratios were calculated using population-averaged generalized estimating equations so that each day of measurement was accounted for, adjusting for maternal education and parity, and infant sex. Data were missing for 12 ^3^, 9 ^4^, 2 ^5^, 16 ^6^, 8 ^7^, 17 ^8^, 3 ^9^, 4 ^10^, 1 ^11^, 7 ^12^, and 6 ^13^ participants.

**Table 4:** Nutrient intake from complementary foods and milk (infant formula and/or breast milk) at 7 and 12 months of age. ^1.^

Nutrient	7 Months			12 Months ^1^		
	Control (*n* = 77)	BLISS (*n* = 85)	% Difference ^3^ (95% CI)	Control (*n* = 70)	BLISS (*n* = 74)	% Difference ^3^ (95% CI)
Energy (kJ) ^2^	2831 (2728, 2938)	2951 (2848, 3057)	5 (−1, 10)	3373 (3179, 3580)	3484 (3339, 3636)	4 (−3, 12)
Protein (g)	16.3 (15.2, 17.5)	17.7 (16.6, 18.7)	8 (−1, 19)	28.5 (26.3, 30.9)	29.4 (27.5, 31.3)	4 (−6, 15)
Protein (% energy)	9.8 (9.4, 10.2)	10.2 (9.8, 10.5)	4 (−2, 10)	14.4 (13.7, 15.1)	14.3 (13.8, 14.9)	0 (−6, 6)
Total fat (g)	33.2 (32.1, 34.3)	35.0 (33.9, 36.1)	**6 (1, 11)**	33.0 (31.0, 35.0)	33.9 (32.3, 35.5)	3 (−5, 11)
Total fat (% kJ)	43.4 (42.2, 44.6)	43.9 (42.7, 45.0)	1 (−2, 5)	36.2 (35.0, 37.3)	36.0 (34.8, 37.2)	−1 (−5, 4)
Saturated fat (g)	14.8 (14.2, 15.4)	15.5 (14.9, 16.1)	5 (−0.2, 11)	15.3 (14.3, 16.3)	14.6 (13.6, 15.7)	−0.5 (−1.9, 0.9)
Saturated fat (% energy)	19.3 (18.6, 20.0)	19.4 (18.8, 20.0)	1 (−4, 6)	16.8 (16.1, 17.4)	15.5 (14.6, 16.5)	**−7 (−14, −0.4)**
Total carbohydrate (g)	78.0 (74.1, 82.1)	79.9 (76.1, 83.8)	3 (−4, 10)	99 (92, 105)	102 (97, 108)	−4 (−13, 6)
Total carbohydrate (% energy)	46.8 (45.9, 47.9)	46.0 (45.0, 47.0)	−2 (−5, 1)	49.7 (48.4, 51.0)	49.9 (48.6, 51.1)	1 (−3, 4)
Added sugars (g)	0.9 (0.6, 1.4)	1.2 (0.9, 1.6)	29 (−20, 108)	6.9 (5.7, 8.3)	5.8 (4.9, 7.0)	−14 (−33, 11)
Added sugars (% energy)	0.6 (0.4, 0.8)	0.7 (0.5, 0.9)	24 (−22, 95)	3.5 (2.9, 4.2)	2.8 (2.4, 3.4)	−17 (−35, 6)
Dietary fiber (g)	2.6 (2.2, 3.2)	2.3 (1.9, 2.8)	−10 (−31, 18)	7.3 (6.5, 8.1)	7.4 (6.8, 8.0)	3 (−10, 18)
Vitamin C (mg)	59.1 (53.9, 64.7)	54.1 (49.8, 58.7)	−10 (−20, 2)	49.4 (44.5, 54.8)	49.6 (44.7, 55.0)	1 (−13, 17)
Vitamin B12 (µg)	0.5 (0.4, 0.6)	0.6 (0.4, 0.7)	13 (−19, 58)	1.1 (1.0, 1.3)	1.2 (1.1, 1.4)	9 (−13, 37)
Calcium (mg)	399 (365, 435)	418 (387, 451)	4 (−8, 16)	556 (502, 616)	562 (513, 615)	1 (−12, 15)
Sodium (mg)	223 (204, 243)	301 (274, 330)	**35 (19, 54)**	666 (613, 722)	711 (663, 762)	8 (−3, 20)

Bold text indicates a statistically significant difference at *p* < 0.05. ^1^ Data presented as geometric mean (95% CI) unless stated otherwise; ^2^ One participant at 12 months in the BLISS group had a very high energy intake (8714 kJ) compared to the rest of the sample because of an extremely high intake of food so was excluded from the analyses at 12 months; ^3^ Difference (95% CI) in nutrient intake shown as percentage difference of BLISS relative to Control adjusted for maternal education and parity, and infant sex.

**Table 5:** Nutrient intake from complementary foods and milk (infant formula and/or breast milk) at 24 months of age. **^1.^**

Nutrient	24 Months		
	Control (*n* = 56)	BLISS (*n* = 57)	% Difference ^2^ (95% CI)
Energy (kJ)	4003 (3812, 4202)	3982 (3806, 4166)	1 (−5, 8)
Protein (g)	37.0 (35.5, 38.6)	37.3 (35.9, 38.9)	2 (−4, 8)
Protein (% energy)	15.7 (15.2, 16.2)	15.9 (15.5, 16.4)	1 (−3, 5)
Total fat (g)	35.7 (34.2, 37.2)	35.1 (33.6, 36.7)	−1 (−7, 5)
Total fat (% kJ)	33.0 (32.0, 34.0)	32.6 (31.7, 33.6)	−2 (−6, 2)
Saturated fat (g)	15.9 (15.0, 16.8)	15.8 (15.0, 16.7)	0 (−8, 8)
Saturated fat (% energy)	14.7 (14.1, 15.3)	14.7 (14.2, 15.2)	−1 (−7, 4)
Total carbohydrate (g)	122 (114, 130)	123 (116, 130)	3 (−6, 12)
Total carbohydrate (% energy)	51.8 (50.5, 53.2)	52.3 (50.9, 53.8)	2 (−2, 6)
Added sugars (g)	16.7 (14.4, 19.4)	14.2 (12.6, 16.1)	−14 (−19, 5)
Added sugars (% energy)	7.1 (6.2, 8.1)	6.1 (5.4, 6.8)	−15 (−29, 2)
Dietary fiber (g)	10.1 (9.3, 10.9)	10.7 (9.9, 11.5)	10 (−1, 22)
Vitamin C (mg)	39.2 (35.5, 43.4)	43.0 (39.0, 47.4)	11 (−4, 28)
Vitamin B12 (µg)	1.7 (1.5, 1.9)	1.7 (1.4, 1.9)	−4 (−19, 15)
Calcium (mg)	619 (562, 681)	610 (556, 670)	−2 (−14, 12)
Sodium (mg)	1123 (1047, 1206)	1090 (1014, 1172)	−2 (−11, 9)

Bold text indicates a statistically significant difference at *p* < 0.05. ^1^ Data presented as geometric mean (95% CI) unless stated otherwise; ^2^ Difference (95% CI) in nutrient intake shown as percentage difference of BLISS relative to Control adjusted for maternal education and parity, and infant sex.

**Table 6:** Sources of energy (kJ) at 7 months of age from complementary foods alone, by food group. ^1.^

	*n* (%) ^2^		Grams ^3^		Energy (kJ) ^4^		
	Control (*n* = 77)	BLISS (*n* = 85)	Control (*n* = 77)	BLISS (*n* = 85)	Control (*n* = 77)	BLISS (*n* = 85)	Difference (kJ) ^5^
Grains and cereals	73 (95)	85 (100)	11 (5, 21)	21 (10, 32)	117 (50, 233)	231 (118, 333)	**127 (62, 192)**
Fruits and vegetables	77 (100)	85 (100)	104 (42, 181)	61 (29, 114)	254 (116, 444)	165 (81, 336)	−67 (−157, 23)
Meat and meat alternatives	61 (79)	82 (96)	4.3 (0.6, 14.0)	10.5 (3.6, 19.4)	35 (4, 107)	71 (22, 144)	**42 (8, 77)**
Milk and milk products	55 (71)	77 (91)	4.4 (0, 21.4)	9.3 (1.7, 23.4)	31 (0, 75)	76 (11, 151)	**49 (17, 80)**
Sweet snacks	34 (44)	58 (68)	0 (0, 1.3)	0.2 (0, 1.7)	0 (0, 21)	3 (0, 28)	2 (−4, 9)
Savory snacks	7 (9)	9 (11)	0 (0, 0)	0 (0, 0)	0 (0, 0)	0 (0, 0)	-
Miscellaneous	63 (82)	80 (94)	42 (10, 94)	27 (10, 59)	9 (1, 31)	40 (15, 83)	**32 (15, 49)**

Bold text indicates a statistically significant difference at *p* < 0.05. ^1^ See Figure 2 for definitions of the nine food groups (including breast milk and infant formula) that foods were assigned to; ^2^ Number (%) consuming each food group at least once over the three days; ^3^ Median (25th, 75th percentile) grams consumed from each food group; ^4^ Median (25th, 75th percentile) kJ consumed from each food group; ^5^ Median difference in kJ from each food group in BLISS relative to Control adjusted for maternal education and maternal parity, and infant sex.

**Table 7:** Sources of energy (kJ) at 12 months of age from complementary foods alone, by food group. ^1.^

	*n* (%) ^2^		Grams ^3^		Energy (kJ) ^4^		
	Control (*n* = 69)	BLISS (*n* = 75)	Control (*n* = 69)	BLISS (*n* = 75)	Control (*n* = 69)	BLISS (*n* = 75)	Difference (kJ) ^5^
Grains and cereals	68 (99)	75 (100)	54 (40, 75)	62 (49, 83)	515 (346, 715)	611 (473, 815)	81 (−32, 195)
Fruits and vegetables	69 (100)	75 (100)	164 (121, 243)	169 (110, 223)	424 (275, 593)	505 (329, 678)	93 (−5, 192)
Meat and meat alternatives	66 (96)	75 (100)	36 (21, 54)	37 (22, 53)	266 (160, 383)	255 (156, 367)	−21 (−94, 53)
Milk and milk products	69 (100)	72 (96)	79 (29, 183)	108 (45, 189)	340 (165, 751)	458 (267, 827)	98 (−74, 271)
Sweet snacks	62 (90)	68 (91)	9.2 (2.2, 18.4)	5.8 (0.7, 15.0)	150 (33, 273)	91 (12, 193)	−30 (−92, 31)
Savory snacks	26 (38)	23 (31)	0 (0, 6.7)	0 (0, 7.5)	0 (0, 66)	0 (0, 79)	-
Miscellaneous	68 (99)	75 (100)	121 (69, 193)	116 (56, 199)	103 (52, 239)	131 (61, 190)	41 (−5, 88)

Bold text indicates a statistically significant difference at *p* < 0.05. ^1^ See Figure 2 for definitions of the nine food groups (including breast milk and infant formula) that foods were assigned to; ^2^ Number (%) consuming each food group at least once over the three days; ^3^ Median (25th, 75th percentile) grams consumed from each food group; ^4^ Median (25th, 75th percentile) kJ consumed from each food group; ^5^ Median difference in kJ from each food group in BLISS relative to Control adjusted for maternal education and maternal parity, and infant sex.
